# Investigation of Delayed Response during Real-Time Cursor Control Using Electroencephalography

**DOI:** 10.1155/2020/1418437

**Published:** 2020-02-08

**Authors:** Hyeonseok Kim, Natsue Yoshimura, Yasuharu Koike

**Affiliations:** ^1^Department of Information and Communications Engineering, Tokyo Institute of Technology, Yokohama, Japan; ^2^Institute of Innovative Research, Tokyo Institute of Technology, Yokohama, Japan; ^3^Precursory Research for Embryonic Science and Technology (PRESTO), Japan Science and Technology Agency (JST), Saitama, Japan

## Abstract

Error-related brain activation has been investigated for advanced brain-machine interfaces (BMI). However, how a delayed response of cursor control in BMI systems should be handled is not clear. Therefore, the purpose of this study was to investigate how participants responded to delayed cursor control. Six subjects participated in the experiment and performed a wrist-bending task. For three distinct delay intervals (an interval where participants could not perceive the delay, an interval where participants could not be sure whether there was a delay or not, and an interval where participants could perceive the delay), we assessed two types of binary classifications (“Yes + No” vs. “I don't know” and “Yes” vs. “No”) based on participants' responses and applied delay times (thus, four types of classification, overall). For most participants, the “Yes vs. No” classification had higher accuracy than “Yes + No” vs. “I don't know” classification. For the “Yes + No” vs. “I don't know” classification, most participants displayed higher accuracy based on response classification than delay classification. Our results demonstrate that a class only for “I don't know” largely contributed to these differences. Many independent components (ICs) that exhibited high accuracy in “Yes + No” vs. “I don't know” response classification were associated with activation of areas from the frontal to parietal lobes, while many ICs that showed high accuracy in the “Yes vs. No” classification were associated with activation of an area ranging from the parietal to the occipital lobes and were more broadly localized in cortical regions than was seen for the “Yes + No” vs. “I don't know” classification. Our results suggest that small and large delays in real-time cursor control differ not only in the magnitude of the delay but should be handled as distinct information in different ways and might involve differential processing in the brain.

## 1. Introduction

In the realm of brain-machine interfaces (BMIs), attempts have been made to decode brain activity to allow the input of commands into BMI systems. Due to its practical advantages, electroencephalography (EEG) has been widely used in BMI systems to infer information about intention to move the upper limb [1], targets and distractors [2], finger movement [3], resting states or motor attempts to move the paretic hand [4], intention to stand or sit [5], and intended direction of movement [6, 7]. Information that is not directly used could still be useful for improving BMI systems.

Passive BMIs exploit implicit information from involuntary brain activity [8]. Likewise, such information can be used to make unsupervised adaptive decoders for BMI systems to improve their performance [9]. Error-related potentials, which occur when an error is made [10], can be used to inhibit the previous command upon detection or to update BMI classifiers through reinforcement learning [11]. As the case may be, the magnitude of error-related potentials reflects the degree of error [12], and several studies have investigated error-related potential in various situations. During a video game task, for example, outcome error and execution error have been classified [13]. Moreover, it has been reported that errors resulting from failures in motor control and sustained attention could be classified and that these errors might involve differential processing mechanisms [14]. A BMI system capable of ignoring command inputs and making corrections when error-related potentials are generated by moving in the opposite direction has also been developed [15]. Even in driving tasks, brain activity associated with discrepancies between cued direction and human intention has been classified [16].

Controlling a cursor in real-time is a common performance task in BMI systems used to execute practical functions such as reaching, which is a common and fundamental action required to perform tasks in daily life. Since errors can take many different forms depending on the BMI system being used and the fact that error-related potentials are task-dependent [17], various kinds of errors can occur when attempting to control a cursor, such as movement in an unintended direction, differences in end points and movement speed, and misclicking; however, response lag to user inputs in BMI systems used in real-time cursor control should be particularly considered. While it is difficult to call the delay an “error,” in this context, the term is related to the performance of the system. Many kinds of errors may result from discrepancies between brain activities associated with the input of a command and the command configuration already programmed into the BMI system; however, lagged responses can occur even if a user supplies the system with suitably relevant inputs. If the user recognizes that the delay is an error and the BMI system learns from this dummy training data, the performance of the system will get worse over time. Moreover, since the user also tries to learn to control the BMI system [18], communication with the system may be prone to failure.

Even though delayed responses can result in communication failure between the user and the BMI system, few studies have investigated these relationships. Errors themselves and error-related potentials that may be similar to response delays have been investigated. Such delayed system responses not only occur in BMI systems but are seen in other systems as well, such as haptic interfaces [19]. It has been reported that in haptic interfacing, task performance can decrease due to delayed responses of the system [20, 21], though the degree to which the delayed response affects performance differs depending on the task [22]. In addition, auditory delays can also affect normal speech [23], which can negatively impact the user's ability to communicate. Thus, delayed responses must also be investigated in BMI systems.

Therefore, the purpose of the study was to investigate how participants respond to delayed cursor control, which is a typical application of BMI systems. Wrist bending was performed to control a cursor, and we investigated brain activity during delayed responses. We divided the delay interval into three groups depending on its length: an interval where participants were unable to perceive the delay, an interval where participants could not be sure whether there was a delay or not, and an interval where participants were able to perceive the delay. Then, we performed two kinds (“Yes + No” vs. “I don't know” and “Yes vs. No”) of binary classifications based on participant's responses and an applied delay (overall, four types of classification). In addition, we identified independent components (ICs) that were associated with high accuracy rates.

## 2. Materials and Methods

### 2.1. Experimental Procedure

Six individuals (five males and one female) with a mean age of 27.0 years (standard deviation: 3.22) participated in the experiment. The lone female participant was left-handed; the other participants were all right-handed. All participants provided written informed consent prior to participating in the experiment. This study was approved by the ethics committee of the Tokyo Institute of Technology (ethics approval number: 2019001), and the experimental protocol was conducted in accordance with the ethical standards outlined in the Declaration of Helsinki.

**Figure 1:**
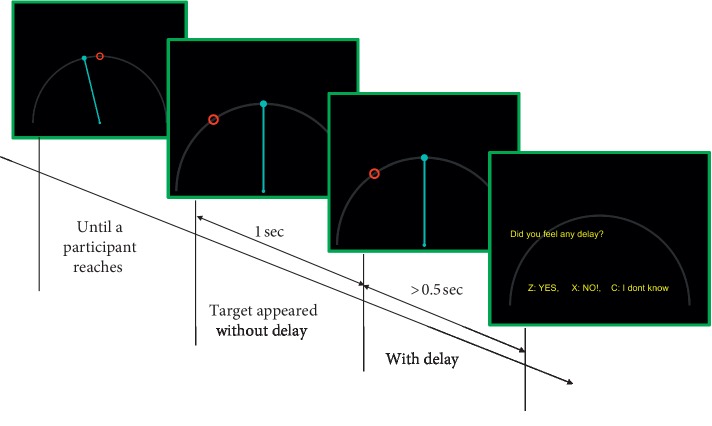
Flow of a single trial. At the beginning of the trial, an initial position indicator (identified by the red circle on the first screen) appeared on the screen to allow the participant to fix the initial position of the movement. When the tracer (cyan pole) reached the initial position indicator, a new target (larger red circle on the second screen) appeared. After 1 s, a delay was applied to the tracer, but nothing changed on the screen. During this stage, the participant performed self-paced reaching. If the participant initialized in the stage without the delay, a “Wait more” message appeared on the screen and data for that trial were discarded. When the tracer reached the target, the target and the tracer disappeared and the participant was asked whether he or she perceived the delay, to which they needed to respond by pressing a corresponding key. This procedure was repeated for each trial.

Before the experiment, a participant sat in a chair in front of a monitor and was allowed to adjust the chair to feel comfortable. The participant was affixed with an electroencephalogram (EEG) cap to which electrodes were attached. Two markers for a motion sensor were attached on the participant's wrist and on a plastic stick that the participant was instructed to hold during the experiment.  Figure 1 shows the flow of a single trial. At the start of the experiment, a red circle, used as an initial position indicator, was aligned in the center of the screen to fix the initial position of movement. The participant controlled the cyan pole (tracer) by bending his/her wrist on the dominant side while holding the stick with the marker. The tracer was moved along the gray arc as shown in Figure 1. When the tracer reached the red circle, another red circle appeared on the screen as a target. The target was positioned to allow participants to access it by bending their wrists by 35 degrees; this angle was selected to allow participants to bend their wrists easily for an extended duration and to allow for the greatest possible range of motion. The target appeared on the left side of the screen for the right-handed participants and on the right side for the left-handed participants. The participants were instructed to wait at least 1.5 s before reaching the target because the delay was applied to the tracer after 1 s. The timing of the delay was selected to make it more difficult for the participant to know whether the delay was applied or not as the tracer reached the initial position indicator. The duration of the delay was between 0 ms and 200 ms; the delay changed between runs by 20 ms intervals. When the participant reached the target, the tracer and the target disappeared, and a question appeared on the screen asking whether the participant perceived the delay or not. When the participant was sure there was a delay, the participant was instructed to press the key to indicate “Yes.” When the participant was sure there was no delay, the participant was instructed to press the key to indicate “No.” When the participant was not sure whether there was a delay or not, the participant was instructed to press the key to indicate “I don't know.” When the participant pressed the corresponding key to respond, the initial position indicator appeared for the next trial. This procedure was repeated so that all participants performed five runs, each consisting of 140 trials; therefore, each participant repeated trials ten times for each delay between 20 ms and 200 ms; they then repeated trials 40 times per run without the delay. The trials were presented in pseudo-random order, with a rest between runs.

### 2.2. Data Acquisition and Preprocessing

The angle data of the wrists were calculated from the positions of the two markers on the wrist and the stick. Each position was measured using the Optotrak Certus motion capture system (NDI, Inc., Waterloo, Canada) and sampled at 100 Hz. Based on the international 10–20 system, EEG signals were measured from 64 channels (Fp1, Fp2, Fpz, AF3, AF4, AF7, AF8, AFz, F1, F2, F3, F4, F5, F6, F7, F8, Fz, FT7, FT8, FC1, FC2, FC3, FC4, FC5, FC6, FCz, C1, C2, C3, C4, C5, C6, Cz, T7, T8, TP7, TP8, CP1, CP2, CP3, CP4, CP5, CP6, CPz, P1, P2, P3, P4, P5, P6, P7, P8, P9, P10, Pz, PO3, PO4, PO7, PO8, POz, O1, O2, Oz, and Iz) using the ActiveTwo system (BioSemi, Amsterdam, The Netherlands), sampled at 2,048 Hz.

EEGLAB [24] was used for preprocessing of the EEG signals, which were re-referenced based on an average reference value and filtered using a band-pass filter (1–40 Hz). Epochs were then extracted from 1 s before the onset of the movement to 1 s after the onset. Epochs in noisy trials and trials where the participant did not wait at least 1.5 s after the target appeared were rejected. We performed independent component analysis to obtain independent electrical sources using the extended Infomax algorithm in EEGLAB [25]. ICs related to noise were rejected.

### 2.3. Classifications

Four types of binary classification were employed. Two classifications were based on the participants' responses (“Yes + No vs. I don't know” and “Yes vs. No”); the other classifications were based on the actual delays. Since the threshold to detect delays varies among individuals, we classified participants into three classes: a class where the participant detected a delay in most of the trials (“Yes”), a class where the participant did not detect a delay in most of the trials (“No”), and an unsure class (“I don't know”). Classifications based on actual delay times were also employed for “Yes + No” vs. “I don't know” and for “Yes vs. No.” Table 1 shows the duration of the delay for each class. The interval was determined by the selection rate for each answer for each participant (see the Results section).

Linear discriminant analysis (LDA) classifiers were used to generate four types of binary classifications. Classifiers were implemented using the Statistics and Machine Learning Toolbox in MATLAB (MathWorks, Inc., Natick, MA, USA). During a period from the onset of the movement to 1 s after the onset, the time series of the remaining ICs were used for analysis. Since a high sampling frequency for EEG signals generates too many factors, the data were downsampled to 100 Hz to reduce computation loads. We used one IC for each classification to determine the relative contribution of each for detecting delays; thus, the ICs could be compared by performance within a classification. Since each IC was used for each classification, as was the last 1 s of each epoch, 100 features were fed into each classifier. The performance of each classifier was assessed using five-fold cross validation. For each fold, the dataset for each participant was partitioned into five smaller datasets with the same number of datapoints using the Statistics and Machine Learning Toolbox in MATLAB. The last remaining dataset had a different number of datapoints when the dataset was not divided by five, without a remainder.

## 3. Results

**Figure 2:**
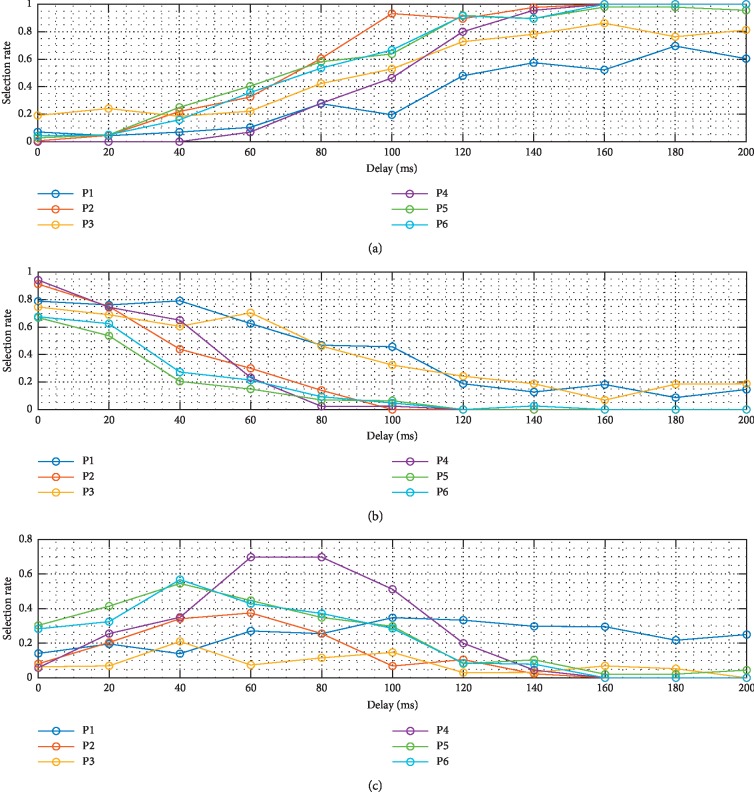
Individual selection rates based on the duration of the delay. Each plot represents a “yes” response (a), a “no” response (b), and an “I don't know” response (c). The selection rate for each response is plotted against the duration of the delay (ms).

Figure 2 displays individual selection rates based on the duration of the delay. For most of the participants, a delay threshold had to be reached before they became confident that they perceived the delay. However, participant 1 showed a consistent selection rate for “I don't know” at all delay intervals, resulting in a lower selection rate in trials with a long delay compared to other participants. All participants, except for participant 1, were more unsure of the existence of a delay in trials with short delays of 40–100 ms compared to other delay durations. The selection rate for the “I don't know” response was the highest for participant 4 for most delay intervals, while participant 3 exhibited the lowest selection rate for this response for most delay intervals.

**Figure 3:**
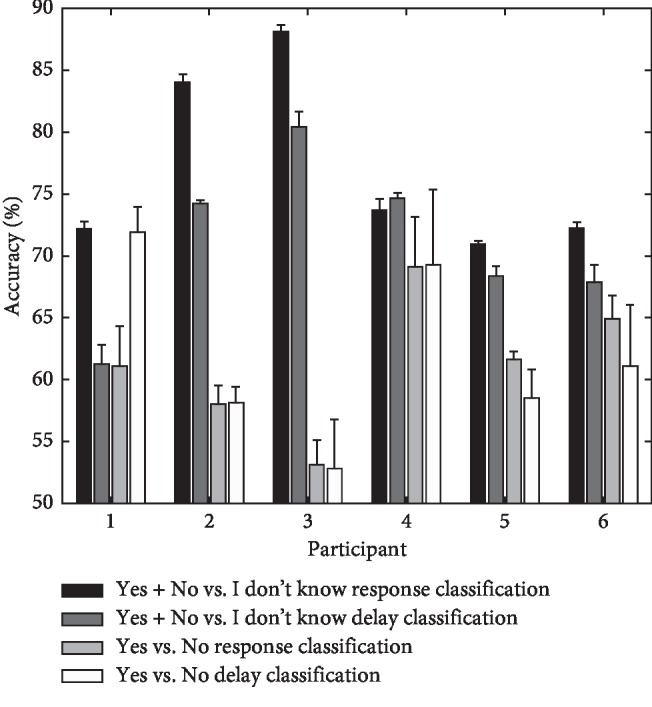
Mean accuracy (%) based on response and delay classifications. The accuracy was averaged across the top five ICs that achieved the highest accuracy rates. Response classification was based solely on each participant's response, regardless of the actual delay duration. Delay classification was based on the delay duration, regardless of participant's response. Each class for the delay classification was determined based on the selection rate for each participant (refer to Table 1).

Figure 3 shows mean accuracy rates based on response and delay classifications. For most of the participants, the “Yes + No” vs. “I don't know” response classification was associated with the highest accuracy compared to other types of classifications (*p* < 0.01 for participants 2, 3, 5, and 6 based on a *t*-test comparing the “Yes + No” vs. “I don't know” response classification and the “Yes + No” vs. “I don't know” delay classification). For participant 4, the accuracy of the “Yes + No” vs. “I don't know” response classification and the “Yes + No” vs. “I don't know” delay classification was similar (*p* > 0.05 for participant 4, based on a *t*-test comparing the “Yes + No” vs. “I don't know” response classification and the “Yes + No” vs. “I don't know”delay classification). Participant 1 showed the higher accuracy for the “Yes vs. No” delay classification, while the other participants showed lower accuracy for the “Yes vs. No” delay classification than the other kinds of classifications. Moreover, accuracy for the “Yes + No” vs. “I don't know” response classification and the “Yes vs. No” delay classification was similar for participant 1 (*p* > 0.1).

**Figure 4:**
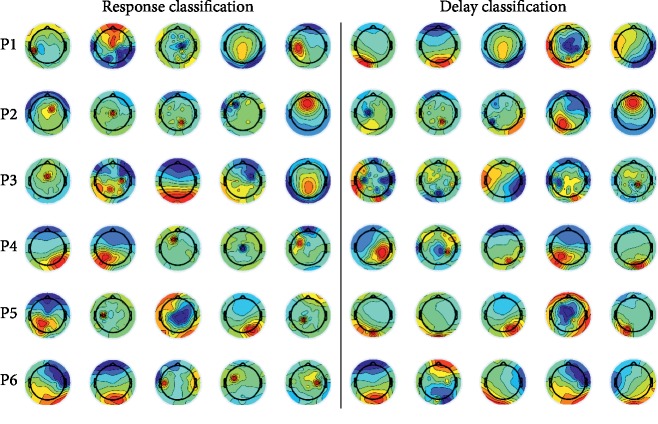
The top five independent components (ICs) that achieved the five highest accuracy rates for the “Yes + No” vs. “I don't know” classification. Each row represents the ICs for each participant (P1–P6). The five ICs in the left grouping are for the response classification, and the five ICs in the right grouping are for the delay classification. The accuracy rates of the five ICs are ranked in the order of decreasing accuracy, moving from left to right.

**Figure 5:**
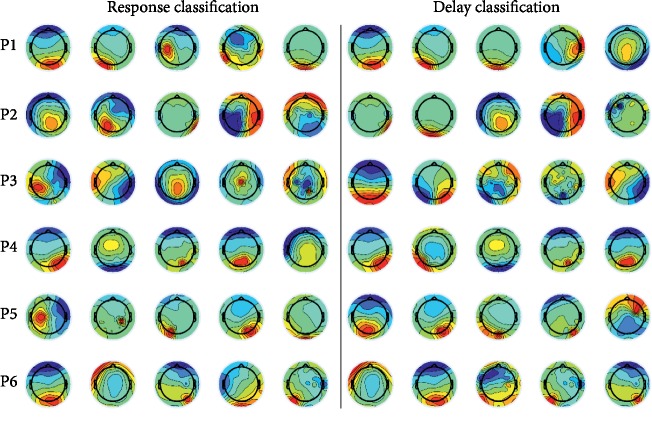
The top five independent components (ICs) that achieved the five highest accuracy rates for the “Yes vs. No” classification. Each row represents the ICs for each of the six participants (P1–P6). The five ICs in the left grouping are for the response classification, and the five ICs in the right grouping are for the delay classification. The accuracy rates of the five ICs are ranked in the order of decreasing accuracy, moving from left to right.

We investigated which ICs contributed to the highest accuracy rates for each type of classification.  Figure 4 shows the top five ICs that achieved the highest accuracy for the “Yes + No” vs. “I don't know” classification.  Figure 5 shows the top five ICs that achieved the highest accuracy for the “Yes vs. No” classification.

## 4. Discussion

In this study, we performed two types of binary classifications (“Yes + No” vs. “I don't know” and “Yes vs. No”) based on the participants' responses and the duration of the delays. For most participants, the “Yes vs. No” classification was associated with higher accuracy than the “Yes + No” vs. “I don't know” classification. That is, classifying participants' confidence in their response, regardless of a delay, was easier than classifying based on whether there was a delay or not. From a BMI system's viewpoint, our results imply that in cases where brain activity can be affected by a delay in the BMI system, short delays do not allow a user to be sure whether there is a delay or not; these short delays may be more problematic, as longer delays provide enough time to perceive the lag. For the “Yes + No” vs. “I don't know” classification, most participants displayed higher accuracy in the response classification than in the delay classification. For all participants, selection rates were below 100% for intervals where they responded with “I don't know,” which means that participants' responses were different even between trials with the same delay. The performance for the “Yes + No” vs. “I don't know” classification based on actual delay times was lower than for the “Yes + No” vs. “I don't know” response classification. In addition, our results show that a class solely for “I don't know” largely contributed to these differences, indicating that some information associated with uncertain responses, rather than delay times, might be represented by information processing in certain areas of the brain. Since participants might think they cannot control the cursor when there is a significantly large delay, “Yes vs. No” might be classified by a sense of agency [26], as ICs are associated with brain areas related to a sense of agency, such as the presupplementary motor area [27], the parietal-premotor network [28], and the inferior parietal areas [29]. For “No” and “I don't know” responses, participants might think they have a sense of agency, but in cases where they respond with “I don't know,” they might experience poor control performance even though they can control the cursor by themselves.

Many ICs that showed high accuracy in the “Yes + No” vs. “I don't know” response classification were related to areas of the brain ranging from the frontal to the parietal lobes, and some of these signals were narrowly localized in the brain, as shown in Figure 4. At the same time, many ICs that showed high accuracy in the “Yes vs. No” classification were related to areas of the brain ranging from the parietal to the occipital lobes, with broader cortical localization compared to the areas associated with the “Yes + No” vs. “I don't know” classification, as shown in Figure 5. The medial frontal cortex plays an important role in monitoring performance outcomes [30]. It has been reported that the frontal midline in the theta band is strongly associated with error-related negativity [31], and the medial prefrontal cortex, which modulates error-related processing, communicates with the lateral prefrontal cortex to comprise a network for action monitoring [32]. In addition, it has been reported that brain activity in the anterior cingulate cortex is related to processing of error-related information [33]; this area is activated not only by the error itself but also by correct outcomes in situations where repetitive errors can be anticipated [34]. These previous studies may support our results, which showed ICs related to the areas associated with the “Yes + No” vs. “I don't know” response classification, reflecting how uncertain delay is perceived for a user of a BMI system. For the “Yes vs. No” classification, few ICs that achieved high accuracy were related to the frontal or central areas; most of the ICs were observed from the parietal and occipital lobes. These areas are related to the dorsal pathway, which is important for information processing from the primary visual cortex to the posterior parietal lobe [35, 36] that is associated with visual sensory perception [37]. It is difficult to know precisely which functional areas in the brain are related to small delays, since the frontal lobe performs many, varied tasks; for example, the anterior cingulate cortex plays a role in conflict detection, error monitoring [38], and response adaptation [39]. Our results suggest that larger delays may not have any relationship with these functions. Moreover, our results suggest that small and large delays in real-time cursor control differ not only in the magnitude of the delay but may be processed in different ways.

**Figure 6:**
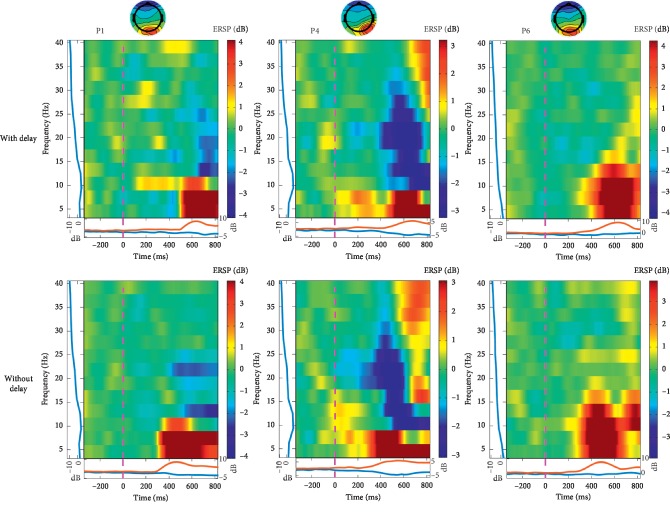
Event-related spectral perturbations of independent components (ICs) related to the processing of visual information from participants 1, 4, and 6. The ICs that achieved the highest accuracy for participants 1 and 4, and the top two highest accuracies for participant 6 based on the “Yes vs. No” delay classification are shown, respectively. The dotted line at 0 s represents the initiation of the movement. The ICs show delayed responses in trials with a delay compared to trials without a delay.

We selected some ICs that achieved high accuracy to investigate how the brain recognizes large delays within trials. We observed event-related spectral perturbations (ERSP) [40] of each IC that achieved high accuracy in the “Yes vs. No” delay classification.  Figure 6 shows ERSPs of certain ICs related to the processing of visual information for participants 1, 4, and 6. The ICs in the trials with a delay exhibited delayed activation compared to the trials without a delay, suggesting that delayed recognition of the onset of the motion is a key factor contributing to the classification. Likewise, since this recognition of the onset of motion had no relation to participants' responses, more ICs that contributed to high accuracy rates were found in the “Yes + No” vs. “I do not know” delay classification than in the “Yes + No” vs. “I don't know” response classification.

In our experiment, most of the participants were unsure of the existence of a delay in trials where the duration of the delay ranged from 40 to 100 ms. However, when we tried to categorize the delays as uncertain and certain, it was difficult to define uncertain delays because participants could respond differently even in trials where the duration of the delay was the same. Instead, the duration of the delay was related to task performance. Point-to-point movement is normally modeled and assessed by Fitts' law [41, 42], which, when fitted with a linearly combined delay term, explained 93.5% of the variance, indicating that task performance may be linearly related to the duration of the delay [43].

We investigated brain activity during delayed cursor control, which can often occur in BMI systems. This can be used to construct intelligent BMI systems to update a decoder online when a BMI system experiences an error. So far, feedforward control has been employed in a BMI system that decodes neural signals to use them as an input command and obtain movement parameters [44]. When a BMI system works well and a user wants to move, feedforward control is sufficient; however, when the system experiences an error because it does not perceive correct user intention, a feedback controller is needed to perform calculations and correct for the delay. This feedback controller is different with a decoder. For real-time control, both the feedforward controller and the feedback controller are components of the system. Input commands generated by the brain are corrected by summing the output of the feedback controller and then transferred as corrected signals, rather than the individual components working in isolation. In this study, we investigated brain activity involved in controlling responses based on the duration of delays. Our results showed that, unlike in general situations when a system is working optimally, when there is a delay between intention to move and the actual movement itself, signals can be generated that are not optimal for feedforward control. Thus, additional systems such as a feedback controller are needed in BMIs; based on our results, commands generated by feedforward and feedback controllers should be separated for the system to work optimally.

## 5. Conclusion

In this study, we confirmed that small and large delays in real-time cursor control might result in differential processing in the brain. However, since the delay may not always be an error, how the delay is related to other kinds of errors should be investigated based on the magnitude of the delay. Understanding these mechanisms may allow for the advanced construction of BMI systems for real-time cursor control in which learning can occur from these errors.

## Figures and Tables

**Table 1:** Delay for each response class based on the delay (unit: ms).

Was a delay felt?	P1	P2	P3	P4	P5	P6
No (not felt)	0–70	0–30	0–70	0–50	0–30	0–30
I don't know (not sure)	70–170	30–90	70–110	50–110	30–110	30–110
Yes (felt)	170–200	90–200	110–200	110–200	110–200	110–200

## Data Availability

The data used to support the findings of this study are included within the article.
